# Are Urethral Pressure Profile Measurements Effective in Diagnosing Urodynamic Stress Incontinence in Women Presenting with Stress or Mixed Urinary Incontinence? Results from a Cross-Sectional Study

**DOI:** 10.3390/medicina61071206

**Published:** 2025-07-01

**Authors:** Konstantinos Pantazis, Themistoklis Mikos, Sofia Tsiapakidou, Iakovos Theodoulidis, Stamatios Petousis, Konstantinos Dinas, Antonio Schiattarella, Antonio Simone Laganà, Apostolos P. Athanasiadis

**Affiliations:** 1 Urogynecology Unit, 2nd Department of Obstetrics & Gynecology, Aristotle University of Thessaloniki, Hippokration General Hospital, 546 42 Thessaloniki, Greece; petousisstamatios@gmail.com (S.P.); dinas@auth.gr (K.D.); 2 Urogynecology Unit, 1st Department of Obstetrics & Gynecology, Aristotle University of Thessaloniki, Papageorgiou General Hospital, 564 29 Thessaloniki, Greece; themis.mikos@gmail.com (T.M.); sofiatsiapakidou@gmail.com (S.T.); iakwbostheo@gmail.com (I.T.); 3 King’s College Hospital NHS Foundation Trust, London SE5 9RS, UK; aschiattarella@gmail.com; 4 Unit of Obstetrics and Gynecology, “Paolo Giaccone” Hospital, Department of Health Promotion, Mother and Child Care, Internal Medicine and Medical Specialties (PROMISE), University of Palermo, 90127 Palermo, Italy; antoniosimone.lagana@unipa.it; 5 3rd Department of Obstetrics & Gynecology, Aristotle University of Thessaloniki, Hippokration General Hospital, 546 42 Thessaloniki, Greece; apostolos3435@gmail.com

**Keywords:** stress incontinence, MUCP, urethral length, urodynamics

## Abstract

*Background and Objectives*: This study aims to evaluate the relevance of urethral pressure profile (UPP) measurements in the diagnosis of urodynamic stress incontinence (USI) in women with stress and mixed urinary incontinence (SUI and MUI). *Materials and Methods*: A cross-sectional chart review was used. All patients who had urodynamic studies (UDSs) in the urogynecology unit of an academic hospital over the last 6 months and complained of SUI or MUI were analyzed. Clinical examination included prolapse grading with the POP-Q system. The presenting symptoms, initial diagnosis before UDS, and results from flow studies—cystometrography (CMG), which included a 1-3-5 cough test at 300–350 mL bladder filling, and urethral pressure profilometry (UPP)—were recorded. *p* < 0.05 was considered significant in all statistical comparison tests; receiver operator characteristic (ROC) curves were also used to determine the best predictor of SUI diagnosis. *Results*: In total, 57 women were included in this study, with a mean age of 60.7 (±9.3). Upon UDS, 28 women (49.1%) demonstrated USI (Group 1), while 29 women (50.9%) did not demonstrate USI (Group 2). No differences between the two groups were noted during free uroflowmetry and the filling phase of CMG. However, the women in Group 2 had a significantly lower MUCP, FUL, and post-void residual after pressure flow compared to the women in Group 1 (*p* = 0.038, 0.003, and 0.04, respectively, upon Student’s *t* test for independent parameters). The ROC analysis indicated that when using MUCP and FUL for the diagnosis of USI, the AUCs are 0.663 (0.525–0.782) and 0.756 (0.623–0.861), respectively. *Conclusions*: By exhibiting correlations between low MUCP/FUL and USI, UPP appears to be a valid test for USI. The value of UPP in diagnosing USI in those with SUI and MUI appears to be clinically important. Further studies are needed in non-SUI patients, in addition to SUI subgroups and various incontinence treatment groups.

## 1. Introduction

Female urinary incontinence (UI), which is a term for the involuntary leak of urine, is a common and bothersome condition that may affect up to one in three individuals over their lifetime [1,2]. Diagnosis is fairly easy via history and physical examinations; however, appropriate categorization may be challenging, particularly when deciding upon treatment options, which may range from behavioral treatment and medication to surgical treatment with or without synthetic grafts. Surgical treatment, which is the most invasive option, is mainly reserved for those with stress urinary incontinence (SUI). Moreover, in “stress-predominant” mixed urinary incontinence, surgical treatment is sometimes offered when other options fail to improve symptoms. As the reproduction of symptoms is often unreliable during examination, it remains desirable for clinicians to diagnose stress urinary incontinence not only clinically but also via special examinations and objective indices.

Because urinary continence in women is a multifactorial function, choosing which objective indices to use is difficult. Continence relies on the adequate and low-pressure filling capacity of the bladder and an effective urethral sphincteric mechanism. In SUI, an increase in the vesical/abdominal pressure exceeds endo-urethral pressure, thus allowing for urine leakage. The sphincteric mechanism of the urethra is complicated. Muscles, nerves, mucosas, and fascias contribute to this function, which relies on the presence of a healthy and functioning striated sphincter controlled by pudendal innervation, a well-vascularized urethral mucosa and sub-mucosa, a properly aligned and functioning intrinsic urethral smooth muscle, and intact urethro-vaginal support. It is not fully understood how these contributors relate to each other in continence and incontinence [3]. However, there is evidence that a weak urethral sphincter and disruption in the supportive mechanism of the bladder and urethra are both involved in the pathophysiology of UI, especially SUI [3,4].

As for the bladder and urethral support, theories have evolved from the “Integral Theory” of Petros and Ulmsten [5]—which shed light on the role of pelvic fascias, ligaments, and connective tissues—and the “Urethral Hanging Theory” [6]—which focused on the downward movement of the urethra below the levator ani plate—to today’s most comprehensive and inclusive “Hammock Hypothesis”, which evolved from DeLancey’s early and later publications [7,8], emphasizing the supportive role that the vagina offers to the urethra during an increase in the abdominal pressure. The vaginal support, in turn, relies on all constructive features of the pelvis, including the endopelvic fascia, the arcus tendineus fascia pelvis, the pubo-urethral ligaments, the levator ani, and the pubococcygeus muscles, and their innervation. Urethral support can be evaluated using subjective methods—such as the pelvic floor’s muscle contraction strength against the examiner’s finger—or indirect methods—such as the ultrasound or MRI of the urethra and levator ani muscles.

The main component of the urethral sphincter is believed to be a striated muscle surrounding the mid-third of the urethra, which is often referred to as the urethral rhabdosphincter; this has already been described in the early 20th century [9]. The most caudal part of this muscle integrates with the fibers of the levator ani. The rhabdosphincter provides intraluminal urethral pressure due to its basic tone, and it can also provide increased pressure via voluntary contraction with the levator ani and all pelvic floor muscles. The intraluminal pressure of the urethra can be directly measured via urethral pressure profilometry—a test that is part of urodynamic studies. A water-filled catheter connected to a pressure transducer or a solid-state transducer catheter is inserted into the urethra and pulled steadily outwards, thus providing pressure measurements along the urethra. The measurements obtained from this test are objective and only marginally depend on the patient’s cooperation, as the only requirement is for the patient to stay still and not contract their pelvic floor muscles. However, this examination has not found widespread acceptance because there are difficulties with the standardization of the technique and conflicting literature evidence; this test also requires highly experienced personnel [10].

This study aims to define the value of urethral pressure profilometry in diagnosing urodynamic stress incontinence (USI) in women.

## 2. Materials and Methods

Cross-sectional chart review: The clinical records of all women who had urodynamic studies (UDSs) for stress or mixed urinary incontinence (SUI or MUI)—in the urogynecology unit of an academic hospital over a control interval of 6 months—were reviewed.

Basic demographics were obtained through a standardized history data sheet, and a complete obstetric, gynecologic, and urogynecologic history was obtained. The data from the International Consultation on Incontinence Questionnaire—Urinary Incontinence Short Form (ICIQ-UI SF) were used to screen patients for inclusion in this study [11,12]. A patient was diagnosed as having SUI if they provided a positive answer in one of the following questions included in the 4th item of ICIQ-UI SF: (i) leaks when coughing or sneezing, and (ii) leaks when physically active/exercising. Patients were diagnosed as having UUI if they provided a positive answer to the following experiences: (i) leaks occurring before making it to the toilet or (ii) leaks occurring when finished with urinating. Patients with SUI-only diagnoses were included; those with UUI-only diagnoses were excluded; those with both SUI and UUI were included if they had additionally answered that SUI symptoms were more bothersome than UUI symptoms. We excluded patients with urge-predominant MUI in order to maintain a more relevant USI-prone sample, acknowledging the sample size’s limitations.

Measurements from the Pelvic Organ Prolapse Quantification (POPQ) system were used to evaluate the severity of any co-existent utero-vaginal prolapse in patients [13]. All women with prolapse greater than Grade I in any vaginal compartment were excluded in order to avoid the confounding factor of advanced prolapse; in particular, we excluded occult incontinence—a condition that is notoriously difficult to reliably diagnose. Similarly, patients who had previous incontinence surgery and those on active treatment for overactive bladder symptoms were excluded (Table 1).

Data from the patients’ multichannel urodynamics (UDSs) reports, which were performed using a Solar Blue Urodynamics System (Medical Measurement Systems-MMS, Enschede, The Netherlands), were recorded. All UDSs were performed according to the ICS Good Urodynamic Practices, and the reports were documented according to IUGA guidelines [14]. All patients presented for UDSs with a recent negative urinary culture and a full bladder. During free uroflowmetry, the patients were asked to void in a uroflowmetry commode, also permitting adaptive positioning if required. The flow rate indices and the voided volume results of the examination were recorded; specifically, the average flow rate (Qave), peak flow rate (Qmax), time to Qmax, and voiding time from each patient were documented, in addition to the voiding pattern. The post-void residual (PVR) was measured via catheterization.

Urethral pressure profilometry was performed in the supine position after free uroflowmetry. A triple-lumen 9-Fry UPP urodynamics catheter (Medical Measurement Systems-MMS, Enschede, The Netherlands) was inserted into the bladder, with the distal opening (bladder pressure line) resting well inside the bladder, and the proximal opening (urethral pressure line) sitting just beyond the bladder neck. The filling line was fed with normal saline at a flow of 60 mL/h. The bladder and urethral lines were filled with normal saline and connected to the UDS machine transducers. A mechanical puller was used to withdraw the catheter at a 1 mm/s rate in order to allow pressure recording along the urethra. The maximum urethral closure pressure and functional urethral length were recorded in each patient’s report.

Filling cystometry was performed after UPP using the same triple-lumen 9-Fry UPP catheter at a filling rate of 100 mL/min. Bladder pressure (Pves) and abdominal pressure (Pabd) were calibrated to the level of pubic symphysis and zeroed to atmospheric pressure. During cystometry, the filling volumes at first sensation (FS), first desire to void (FDV), normal desire to void (NDV), strong desire to void (SDV), and maximum cystometric capacity (MCC) were obtained. A cough test was performed with a bladder volume of 300–350 mL. It is noted that the higher the bladder filling volume, the more likely a stress test is to be positive; however, there is a risk that this test cannot be completed in patients with reduced bladder capacity. On the other hand, a stress test at low filling volumes has reduced sensitivity. Our standard practice is to perform stress tests at 300–350 mL, which is within the ICS-recommended volume of 200–400 mL, in order to establish a diagnosis of USI [15,16]. Additionally, a single cough test was performed during the beginning and at 50–100 mL intervals as a standard quality control, and this inherently ensures that severe incontinence at low filling volumes will not be missed.

A diagnosis of detrusor overactivity (DO) was confirmed if there was an increase in the Pves trace without an increase in Pabd and a sensation of urge to urinate, whether after provocation or spontaneously. Upon voiding cystometry (pressure flow study), the pre-micturition Pdet, voided volume, maximum flow rate (Qmax), time to Qmax, voiding time, and maximum voiding pressure were documented.

Selected urodynamic indices were compared against the final urodynamic diagnosis (USI/no USI). *p* < 0.05 was considered significant in all statistical comparison tests. ROC curve analysis (receiver operating characteristic) was performed, and the AUC (area under the curve) value was estimated in order to assess the correlation between UPP indices and USI (MedCalc Software v19.1, Mariakerke, Belgium).

## 3. Results

**Figure 1:**
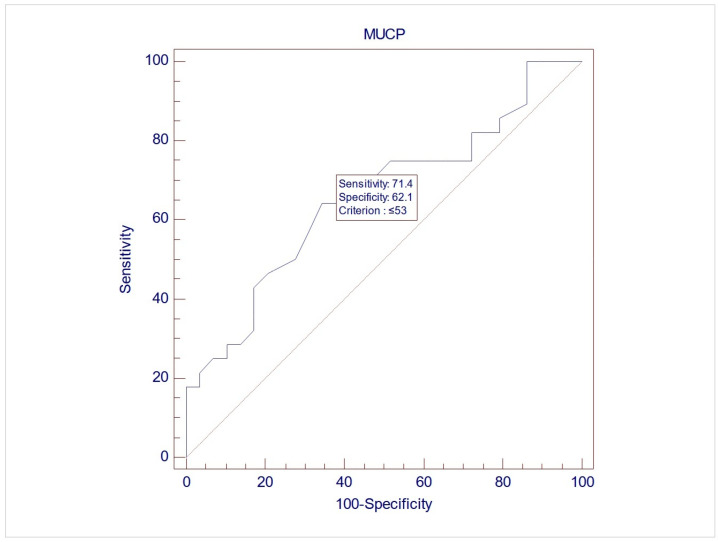
ROC graph of MUCP against USI diagnosis. AUC (area under the curve) = 0.663 (95%CI 0.525–0.782).

**Figure 2:**
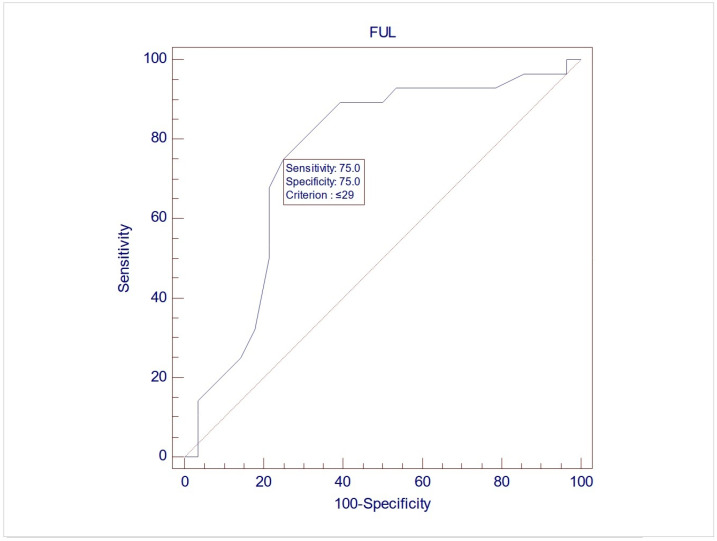
ROC graph of FUL against USI diagnosis. AUC (area under the curve) = 0.756 (95%CI 0.623–0.861).

**Figure 3:**
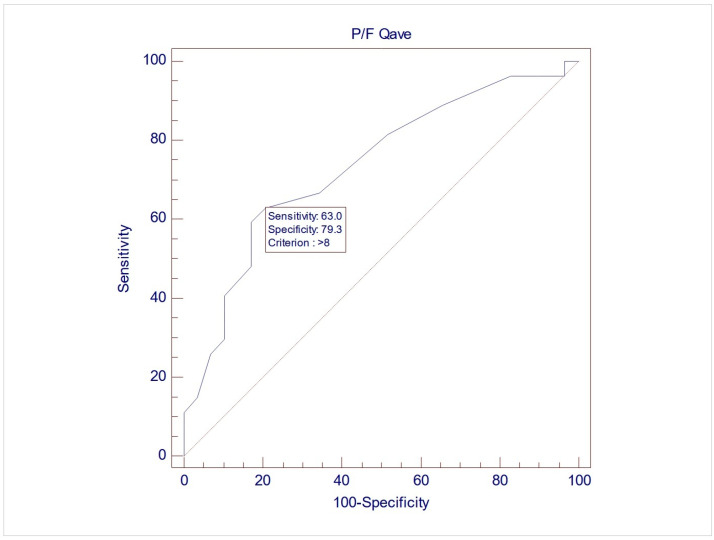
ROC graph of Qave against USI diagnosis. AUC (area under the curve) = 0.734 (95%CI 0.599–0.843).

The records of 307 women were screened for inclusion, and eventually, 57 women were included in this study, with a mean age of 60.7 (±9.3). Upon UDS, 28 women (49.1%) demonstrated USI (Group 1), while 29 (50.9%) did not demonstrate USI (Group 2). No differences between the two groups were noted during free uroflowmetry and the filling phase of CMG. However, the women in Group 2 exhibited significantly lower MUCP, FUL, and post-void residuals after pressure flow compared to women in Group 1 (*p* = 0.038, 0.003, and 0.04, respectively, via Student’s *t* test for independent parameters) (Table 2). The ROC analysis indicated that when using MUCP and FUL for the diagnosis of USI, the AUCs are 0.663 (95%CI 0.525–0.782) and 0.756 (95%CI 0.623–0.861), respectively (Figure 1 and Figure 2). It appears that FUL measurements were the most accurate diagnostic metrics for USI confirmation. The average flow rate (Qave) also exhibited correlations relative to the diagnosis of USI with an AUC of 0.734 (95%CI 0.599–0.843), although this cannot be logically considered a diagnostic parameter (Figure 3).

## 4. Discussion

Urethral function tests have not found their way to routine clinical practice over time, despite the obvious clinical need to evaluate urethral function in women with UI. Part of this problem is the lack of appropriate standardization, as rightfully noted by the fifth ICI Committee (urodynamic testing) during the 7th International Consultation on Incontinence, which was held in 2021 [3]. Indeed, previously in 2016, the sixth ICI Committee (urodynamic testing)—during the 6th International Consultation on Incontinence [17]—recommended that Valsalva leak point pressure (VLLP) and urethral closure pressures should not be used as a single factor to grade the severity of UI, and they also did not recommend the use of urethral function tests for predicting the outcome of any surgical treatment for SUI (Grade C). However, later studies indicated that the ability of such tests to correlate with patient-filled questionnaires and clinical diagnosis improves with increased bladder filling upon testing [18]. Moreover, in other studies, the severity of patient symptoms correlated with low scores during urethral tests; this is compatible with the diagnosis of intrinsic sphincter deficiency (ISD) [19,20]. Therefore, there appears to be a link between urethral function tests and USI diagnosis, which has failed to be clarified in early studies.

This study showed that women diagnosed with USI had significantly lower MUCP values than those without USI (51.5 vs. 68.58, *p* = 0.0383), which is the main urethral function index. Lower MUCP values mean weaker sphincteric action, and this, in turn, means that the patient is more likely to suffer from UI. It is difficult to interpret why there is still controversy in the literature given our clear findings. One can speculate that the specimen in this study was more homogeneous than others, as prolapse, urge urinary incontinence (UUI), and UUI-predominant cases were excluded by design. Moreover, referral patterns were homogeneous, as almost all cases were referred by our department’s Urogynecology Outpatient Clinic. The urodynamics technique was homogeneous as well, given that the clinic is a physician-led unit and all examinations were carried out by two trained urogynecologists.

Moreover, an even stronger negative relation between FUL and USI was observed (26.88 in USI vs. 30.89 in non-USI cases, *p* = 0.0037). FUL is an under-investigated index of urethral function. It is practically overshadowed by the MUCP in the literature, which is understandable because MUCP, being a pressure measurement, appears to be more logical as a direct index of urethral function. Urethral length, as measured using ultrasound, has been proposed as a failure predictor for mid-urethral sling (MUS) incontinence operations [21], but the urodynamic functional length of the urethra—measured relative to intraluminal pressure during urethral profilometry—is missing from the literature. Given our findings, FUL might attract more attention in future diagnostic models.

A further interesting finding is the positive relation between the average flow rate (Qave) and USI during pressure flow studies, which denotes the urinating phase at the end of filling cystometry with catheters still in situ. This is difficult to interpret because no such correlation was exhibited during the free flow study (Uroflow), which is the urinating phase at the beginning of the urodynamics study prior to the insertion of the catheters. No explanation can be found in the literature, nor can this study explain this finding. Considering that the female urethra is as short and mobile sphincteric structure, we could speculate that the change in the shape (straightening) and mobility of the urethra because of the catheter could interfere with both basic intrinsic urethral pressure at rest or voiding and urethral reflexes, which normally increase the urethral pressure upon a sudden increase in abdominal pressure [22]. This could be a possible common mechanism for higher voiding flows and lower urethral function upon coughing, which increases the risk of USI. Therefore, this finding should be carefully considered, and further research is needed.

Similarly, a negative relation between the post-void residual (PVR) test and USI was exhibited during the pressure flow study but not during the initial Uroflow test. This finding is also difficult to interpret. Although it follows logically that a “weaker sphincter” will allow for better emptying, this should have been observed during the free Uroflow study, but it was not. The same speculation that the catheter might interfere with the urethral sphincter’s function, as mentioned above, could apply to the lower PVR. Therefore, this intriguing finding should also be taken into careful consideration.

Currently, international research and clinical interest on female SUI rightfully focuses on symptoms and quality-of-life assessment tools (i.e., validated questionnaires), and leak tests, and these are also used in order to decide upon treatment methods. “Dry” tests, such as UPP, have been questioned due to weaknesses in their standardization and reproducibility. This leaves clinically and urodynamically “dry” patients with SUI complaints without objective technical investigation. Indeed, in our study population, which was relatively homogeneous for SUI, only about half of the women (28 vs. 29) were observed with USI. The other “dry” half of female participants had no data on their urethral sphincteric mechanism. Considering that some will not respond to conservative treatment and will return for increased and possibly individualized surgical treatment, we note that it would be useful to have their urethral function tests available to supplement stress tests and questionnaire data during clinical decision-making.

This study appears to benefit from its strict study protocol, the homogeneity of measurements (all examinations were performed by the same clinicians), and the robust study group based on specific inclusion and exclusion criteria. On the other hand, the weight of the results is limited by this study’s retrospective design and the lack of structured patient self-reporting, other than the brief ICIQ-UI SF. This study could benefit from the use of more robust and comprehensive standardized questionnaires, which were not available in the patients’ records. Another weakness of this study is the small sample size. As mentioned above, we opted for the homogeneity of an SUI/SUI-predominant MUI population—comprising 57 cases—compared to the complexity of a more mixed population comprising a total of 307 cases, in order to enhance the effect of the sphincteric function on our results. Moreover, we note that data from treatment outcomes were not included because they were inconsistent, as many patients continued treatment elsewhere. This is an inherent weakness of the retrospective design of this study. Furthermore, as a single-center study, generalization is admittedly further limited. Overall, this study is focused on mere comparisons between urodynamic parameters and the diagnosis of USI in a restricted group of patients who complained of SUI/SUI-predominant MUI.

## 5. Conclusions

In this small retrospective study of a homogeneous population, the UPP was relevant to USI diagnosis in SUI/SUI-predominant women. In particular, MUCP and FUL were lower in women with USI, with fair correlations relative to USI diagnoses. The UPP may be a useful diagnostic addition in women with SUI and in women who fail to reproduce their UI symptoms during clinical evaluation.

Prospective studies in larger populations need to be carried out in non-SUI patients and, additionally, in SUI subgroups and various incontinence treatment groups.

## Figures and Tables

**Table 1:** Inclusion and exclusion criteria.

Exclusion Criteria	Inclusion Criteria
Prolapse stage higher than POP-Q Stage 1	Stress urinary incontinence
Previous incontinence surgery	Mixed urinary incontinence
Active OAB treatment	

**Table 2:** Results: Background and urodynamic results compared via USI/non-USI diagnosis. Q ave: average flow; Qmax: maximum flow; Vol: voided volume; PVR: post-void residual; MUCP: maximum urethral closure pressure; FUL: functional urethral length. (*): *p* ≤ 0.05 via Student’s *t*-test.

	Group 1 (no USI)		Group 2 (USI)			
	*n* = 29		*n* = 28			
	Mean	SD	Mean	SD	*p*	95% CI
Age	59.69	8.95	61.85	9.72	0.385	-
ICI-Q SF score	15.27	4.12	16.4	4.11	0.344	-
Uroflow						
Qave (mL/s)	7.24	6.51	8.44	6.65	0.522	-
Qmax (mL/s)	20.37	21.03	19.72	15.97	0.901	-
Vol (mL)	217.18	165.6	188.63	164.67	0.528	-
PVR (mL)	53.1	97.49	32.64	74.79	0.379	-
CMG						
First desire (mL)	250.59	111.57	276.07	108.54	0.056	-
Normal desire (mL)	403.16	100.62	397.16	123.54	0.851	-
UDS capacity (mL)	559.31	132.77	552.15	66.87	0.802	-
Pressure flow						
Qave (mL/s)	7.24	3.75	11.56	5.98	0.0019 *	1.66–6.97
Qmax (mL/s)	19.45	10.65	23.68	11.41	0.1533	-
PVR (mL)	87.93	95.33	42.14	69.46	0.0435 *	−90.19–−1.38
UPP						
MUCP (cmH_2_O)	68.58	36.03	51.5	23.1	0.0383 *	−33.22–−0.95
FUL (cm)	30.89	5.36	26.88	4.51	0.0037 *	−6.66–−1.36

## Data Availability

The data presented in this study are available on request from the corresponding author. The data are not publicly available due to privacy or ethical restrictions.
